# From ticket to protection: The potential of embedding travel-clinic prompts in Qatar’s airline booking systems

**DOI:** 10.5339/qmj.2026.40

**Published:** 2026-06-16

**Authors:** Lina Rasmi AlMbaidin, Jihene Maaloul, Muna Al Maslamani, Mohammed Abukhattab, Mohamed Iheb Bougmiza

**Affiliations:** 1 Preventive and Community Medicine Residency Program, Hamad Medical Corporation, Doha, Qatar; 2 Preventive and Community Medicine Residency Program, Family and Community Medicine Department, Primary Health Care Corporation, Doha, Qatar; 3 Faculty of Medicine, Sousse University, Sousse, Tunisia; 4 Division of Infectious Diseases, Department of Medicine, Hamad Medical Corporation, Doha, Qatar; 5 Communicable Disease Center, Hamad Medical Corporation, Doha, Qatar; 6 College of Medicine, QU Health, Qatar University, Doha, Qatar

**Keywords:** Travel medicine, pre-travel consultation, digital health informs, airline booking, Qatar, infectious disease prevention

## Abstract

**Background:**

Qatar residents undertake substantial outbound travel, often to destinations with risks of malaria, yellow fever, and enteric infections; however, many travelers remain unaware of preventable health risks or how to access pre-travel care. Local data show high vaccination needs and a rising incidence of imported malaria among non-immunized residents, highlighting a missed opportunity for earlier intervention at the point of booking.

**Methods:**

A narrative synthesis of peer-reviewed travel medicine literature and Qatar-specific epidemiological data was combined with stakeholder and service mapping to identify gaps in current information pathways and define the core features of a digital prompt aligned with national public health services.

**Results:**

Global evidence shows that many international travelers experience travel-related illness, while data from Qatar indicate frequent travel to high-risk destinations and substantial vaccination needs. Current airline and government websites provide only static information and lack contextual prompts, clear next steps, and direct referral pathways. We propose a smart, bilingual travel-health pop-up, triggered only for flagged destinations, that briefly summarizes key infectious disease risks in Arabic and English and directs users to locally aligned World Health Organization/Centers for Disease Control and Prevention (WHO/CDC)-based guidance and national travel and vaccination clinics.

**Conclusion:**

This low-friction, privacy-preserving pop-up could help convert awareness into timely pre-travel consultation in Qatar and serve as a scalable, low-cost complement to existing education channels. A multi-stakeholder pilot study is recommended to assess its feasibility, impact, and scalability.

## 1. INTRODUCTION

Travel-related illnesses, defined as acute or subacute conditions arising during international travel or within 4-6 weeks of return, encompass both infectious diseases - such as diarrhea, malaria, typhoid, and dengue - and non-infectious conditions such as deep vein thrombosis.^1^ These illnesses constitute a significant global health burden, affecting approximately 43-79% of travelers to developing countries, with diarrhea being the most common presentation.^2^ Most patients seek care within one month of returning.^3^ This review focuses specifically on the infectious subset, which is largely preventable yet can become severe if left unaddressed prior to departure.

Recent evidence further confirms this burden. A 2025 meta-analysis of 10 cohort studies (*n* = 8,478) estimated a pooled incidence of travelers’ diarrhea of 36% (95% CI: 24-41%) among travelers to high-risk destinations.^4^ In addition, prospective app-based surveillance found that 35% of international trips were associated with at least one illness episode, with gastrointestinal symptoms occurring in 19% of trips.^5^ Qatar serves as both a major expatriate hub and a growing international tourism and transit destination, resulting in substantial outbound travel to diverse regions worldwide, including many with significant infectious disease risks. A Qatar-based travel clinic study found that residents frequently traveled to high-risk destinations, mainly in sub-Saharan Africa (Tanzania 16.5% and Kenya 15.1%). Others visited Saudi Arabia (12.9%), South Africa (7.9%), Sri Lanka (6.8%), Malaysia (5.4%), Uganda (4.7%), and India (4.3%). Nearly all travelers (97%) required vaccination, with typhoid (69%), tetanus-diphtheria-pertussis (Tdap) (62%), hepatitis A (55%), influenza (49.3%), and yellow fever (39%) being the most commonly administered vaccines.^6^ Approximately one in five travelers (21%) had pre-existing conditions (hypertension or diabetes), which increased their vulnerability to travel-related illnesses. The authors concluded that limited travel medicine services and low awareness - especially among travelers visiting friends or relatives - heighten the risk of travel-related illness.^6^

Another Qatar-based study focused on malaria, referring to it as a significant public health concern because most residents are not immunized;^7^ so any local transmission could cause severe illness and outbreaks. The study reported an increase in imported malaria cases, mainly among travelers returning from India, Nepal, Pakistan, and Sudan. All infections were acquired abroad in malaria-endemic regions.^7^ This pattern was further confirmed by Bansal et al., who documented that malaria cases in Qatar originate predominantly from the Eastern Mediterranean, African, and Southeast Asian regions, all of which substantially overlap with destinations identified as high-risk in international travel-health guidance.^8^ Crucially, the post-COVID-19 rebound in malaria cases, rising from 157 in 2020 to 465 in 2022, was directly attributed to the resumption of travel to endemic countries, with a yearly seasonal peak occurring between July and September, coinciding with expatriates returning from summer visits to their home countries.^8^ Taken together, these data confirm that travel to malaria-endemic and other infectious disease-risk destinations is a frequent and clinically significant pattern among Qatar’s resident population, further underscoring the need for earlier, booking-stage interventions.

## 2. PRE-TRAVEL VACCINATIONS: MANDATORY AND RECOMMENDED IMMUNIZATIONS

Pre-travel vaccinations fall into two equally important categories, and understanding the difference between them is essential for travelers departing from Qatar.

The first category - mandatory vaccinations - includes the yellow fever vaccine, which requires proof of vaccination via the International Certificate of Vaccination or Prophylaxis (ICVP or “Yellow Card”) for entry into multiple countries.^9^ Saudi Arabia additionally mandates quadrivalent meningococcal ACWY vaccination (covering serogroups A, C, W, and Y) for all Hajj and Umrah pilgrims.^10^ Some countries may also require proof of polio vaccination for travelers arriving from poliovirus-affected regions.^11^ As travelers are typically informed of these entry requirements through government or airline channels, mandatory vaccines fall outside the primary focus of this intervention.

The second category - and the primary focus of this intervention - encompasses recommended vaccinations, which are not enforced at borders but are strongly advised based on destination-specific infectious disease risks, the traveler’s planned itinerary, duration of stay, accommodation type, and underlying health status. A comprehensive review of the current evidence base identifies the full spectrum of travel vaccines for international travelers, updated in line with Advisory Committee on Immunization Practices guidance.^12^ For Qatar-based outbound travelers specifically, the most clinically relevant recommended vaccines include: typhoid vaccination for travel to South and Southeast Asia and sub-Saharan Africa; hepatitis A vaccination, which is universally advised for travel to low- and middle-income countries; hepatitis B vaccination for longer stays or anticipated healthcare exposure; rabies pre-exposure prophylaxis for rural or adventure travel itineraries; Japanese encephalitis vaccination for rural travel across Asia; and cholera vaccination for travel to active outbreak settings or humanitarian work contexts.^12^

## 3. STAKEHOLDERS

The national carrier, Qatar Airways (QA), connects Doha to an extensive network of international destinations, while Hamad International Airport consistently ranks among the world’s busiest aviation hubs, together facilitating substantial cross-border passenger movement. This level of global connectivity is relevant from a public health perspective, as it increases population mobility and potential exposure to travel-related health risks. While the airline’s travel-requirements page links to authoritative health guidance from the World Health Organization (WHO), Centers for Disease Control and Prevention (CDC), and Qatar’s Ministry of Public Health (MOPH), rather than providing the advice itself,^13^ these links appear only within the requirements section and are easy to overlook. There is no contextual pop-up when a traveler selects a destination with infectious disease risk, and there are no immediate “what to do now” actions. The proposed service addresses this gap by introducing a timely and visible prompt with clear next steps at the point of booking.

As Qatar’s central health authority, the Ministry of Public Health is responsible for disease surveillance, traveler-health regulations, and the issuance of official travel-health advisories. Qatar’s national healthcare system is led by two primary government agencies: Hamad Medical Corporation (HMC) - the nation’s leading public hospital network and tertiary-care provider with over 2,000 beds^14^ - and the Primary Health Care Corporation (PHCC) - which serves approximately 1.6 million people as the state-run primary-care provider.^15^ For clinical referrals, government-run services include Hamad Medical Corporation’s Travel Clinic at the Communicable Disease Center in Doha, which provides pre-travel consultations, post-travel care, and a wide range of travel-related vaccinations and prophylaxis services.^6^ In addition, specialized travel vaccination services are available through designated travel clinics within the public healthcare system, including the PHCC.^6^

## 4. HOW THE SUGGESTED SERVICE WORKS: SMART TRAVEL-HEALTH POP-UP, FROM ALERT TO ACTION

This concept would deliver a contextual, bilingual travel-health prompt at the passenger-details step. A lightweight, dismissible pop-up would appear only when a traveler selects a flagged destination. A destination would be automatically flagged as high-risk when the selected country meets predefined travel-health risk criteria based on internationally recognized travel-health guidance, including the World Health Organization (WHO) International Travel and Health recommendations and the Centers for Disease Control and Prevention Travel Health Notices, which are commonly used in travel medicine to classify destination-related health risks and vaccination recommendations for international travelers.^16,17^ Guidance presented in the pop-up could be drawn from WHO and CDC Traveler’s Health resources, then lightly adapted by Qatar public-health experts to align with local policy, with artificial intelligence (AI)-assisted periodic updates. When an outbreak is noted (e.g., dengue), the prompt includes a plain-language precaution (e.g., mosquito protection). Arabic and English were selected as the default languages due to Arabic being Qatar’s official language and the extensive use of English in public communication.^18^ These languages will be used during the pilot phase, with the potential to expand to additional languages in subsequent phases. The core message consists of two concise lines shown together for clarity:

“Your trip may involve infectious disease risks (e.g., malaria, yellow fever, typhoid). A quick pre-travel check can help keep you safe.”

“Book a travel clinic visit by phone: PHCC (Hayyak) 107 or HMC (Nesma’ak) 16060. Arabic/English available. Average time: 15–30 min for advice; vaccines as needed.”

Hayyak - Arabic for “Welcome” - is PHCC’s patient appointment and information line, reachable via the short-dial number 107; Nesma’ak - Arabic for “We hear you” - is HMC’s clinical access and support line, reachable at 16060; both are Qatar domestic short codes.

**Figure 1.**
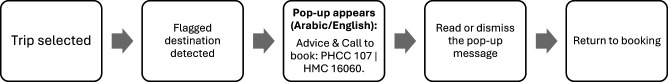
Conceptual model of a travel-health pop-up to be implemented within airline and travel agency booking platforms during the flight booking process.

The experience would appear only once per booking, would be easy to dismiss, and would remember the choice for that session, built on privacy and trust (no medical data collected or stored; links only to government clinics and phone numbers), and designed for accessibility (clear typography, keyboard navigation, and screen-reader compatibility), with a visually engaging layout to capture traveler attention at the point of booking. A simplified view of the trigger, message, and referral options is shown in Figure 1.

## 5. DISCUSSION

Despite Qatar’s documented outbound travel burden and the clear clinical case for earlier intervention, no booking-stage travel-health prompt currently exists within the region - a gap that international experience suggests is both addressable and overdue. Across Europe, longitudinal analysis of EuroTravNet surveillance data evaluated the effect of pre-travel consultation on illness among returned travelers, demonstrating that consultation is clinically meaningful yet consistently underutilized, particularly among those visiting friends and relatives and those with last-minute itineraries. This underscores the need for proactive, earlier intervention pathways that reach travelers before departure.^19^ In Canada, Wilson et al. proposed replacing the paper ICVP with a complementary digital vaccination record, considering that mobile integration could enhance reliability, reduce the risk of loss or damage, and enable direct linkage to appointment-booking systems. However, implementation challenges related to border-crossing verification and cross-jurisdictional interoperability were acknowledged as substantial barriers to adoption.^20^ In the United States, a survey examining knowledge, attitudes, and practices toward mobile travel-health applications found that while 44 commercially available apps offered destination-specific immunization guidance, significant gaps remained in user engagement, clarity of purpose, and alignment with traveler preferences, indicating that the existence of digital tools does not in itself translate into behavioral action.^21^ A participatory surveillance application developed at the Travelers’ Advice and Immunization Center at Massachusetts General Hospital demonstrated the feasibility of collecting real-time health data from travelers across high-risk destinations in Asia, Africa, and the Americas. However, participants had a mean age of 37 years and were predominantly female, limiting the generalizability of the findings to broader, more diverse traveler populations.^22^ At the institutional level, the United States CDC redesigned its Yellow Book travel-health website using a structured user-experience framework, achieving a 46% increase in annual web traffic (exceeding 5.7 million page views) and markedly improved mobile accessibility. Notably, the platform is explicitly oriented toward healthcare professionals as its primary audience rather than travelers themselves, constraining its direct public-facing behavioral impact.^23^

None of these initiatives achieved integration at the point of booking itself - the single touchpoint where the traveler’s destination is known, the decision window is open, and a referral to a travel clinic carries the most immediate clinical value. The proposed Qatar model addresses this gap directly, positioning a single dismissible bilingual prompt at the moment of flight selection and converting awareness into a frictionless referral to HMC and PHCC travel clinics at the earliest and most actionable stage of the travel journey.

## 6. CONCLUSION

Qatar’s substantial outbound travel volume, frequent trips to malaria-, yellow fever-, and enteric-risk destinations, and the documented rise in imported malaria reveal a fixable gap at the point of booking. A one-time, dismissible, Arabic/English pop-up - shown only for flagged trips, built on WHO/CDC guidance adapted to local policy, and directing travelers to HMC and PHCC travel clinics - offers a low-cost, high-impact pathway from awareness to timely protection without collecting medical data or disrupting the booking experience. To move this from concept to delivery, QA, MOPH, HMC, and PHCC should convene a working group to confirm risk triggers, finalize bilingual content, agree on a governance and review structure, and launch a short pilot with shared Key Performance Indicators KPIs: Clinic call volume would reflect referral activity, vaccine uptake would capture completed preventive action, and traveler engagement would measure the prompt’s visibility and acceptability, giving stakeholders a practical dashboard for judging the pilot’s success. The system architecture is platform-agnostic and scalable beyond Qatar Airways to other carriers at Hamad International Airport and online booking platforms, though expansion is reserved pending proof-of-concept validation. Critically, each pop-up would embed direct one-tap access to referral lines 107 and 16060, converting the prompt from a passive display into an immediate, frictionless action.

## DATA AVAILABILITY

No new data were generated or analysed in this study; all data discussed are derived from publicly available, cited sources.

## ETHICAL APPROVAL

Not applicable. This narrative review did not involve human participants, animals, or identifiable patient data.

## AUTHOR CONTRIBUTIONS

L.R.A. contributed to the conceptualization, methodology, literature review, and writing of the original draft, and prepared the figure. J.M. contributed to the methodology, supervision, and critical review and editing of the manuscript. M.A.M. provided infectious-disease and travel-medicine expertise, validated the clinical content, and reviewed and edited the manuscript. M.A. contributed travel-clinic and clinical expertise, validated relevant content, and reviewed and edited the manuscript. M.I.B. contributed to the methodology, supervision, and critical review and editing of the manuscript.

## FUNDING

This research received no specific grant from any funding agency in the public, commercial, or not-for-profit sectors.

## ACKNOWLEDGMENT

The author(s) have no acknowledgements to declare.

## DISCLOSURE OF AI USE

During the preparation of this manuscript, the authors used an AI-based language tool solely for vocabulary refinement and English-language editing. The tool was not used to generate content, analyse data, or produce any scientific conclusions. The authors reviewed and edited all output and take full responsibility for the content of the publication.
